# Supplementary material for: The complex simplicity of the brittle star nervous system
Source: Front Zool. 2018 Feb 1;15:1. doi: 10.1186/s12983-017-0247-4 (PMC5796562; doi:10.1186/s12983-017-0247-4)
Supplement: Supplementary file 6 — All components of the arm nervous system (green – ectoneural system; magenta – hyponeural system; light blue – mixed peripheral nerves) plus the intervertebral muscles (brown) and hydrocoel (red). This is an interactive 3D model generated from the original .blend file (Additional file 2) using the Blend4Web tool (https://www.blend4web.com). The model can be opened in any modern web browser. Upon opening, zoom out using the scroll wheel. To rotate, press and hold the left mouse button. To pan the view, press and hold the right mouse button. (HTML 23,040 kb) [file 12983_2017_247_MOESM6_ESM.html]

Blend4Web Player


0%

dev

blend4web.com
